# Identification of human remains from the Second World War mass graves uncovered in Bosnia and Herzegovina

**DOI:** 10.3325/cmj.2015.56.257

**Published:** 2015-06

**Authors:** Damir Marjanović, Negra Hadžić Metjahić, Jasmina Čakar, Mirela Džehverović, Serkan Dogan, Elma Ferić, Snježana Džijan, Vedrana Škaro, Petar Projić, Tomislav Madžar, Eduard Rod, Dragan Primorac

**Affiliations:** 1 International Burch University, Sarajevo, Bosnia and Herzegovina; 2 Institute for Anthropological Researches, Zagreb, Croatia; 3 Faculty of Science, University of Sarajevo, Sarajevo, Bosnia and Herzegovina; 4 Institute for Genetic Engineering and Biotechnology, University of Sarajevo, Sarajevo, Bosnia and Herzegovina; 5 Genos DNA Laboratory, Zagreb, Croatia; 6 University of Osijek, Medical School, Osijek, Croatia; 7 University of Zagreb, Medical School, Zagreb, Croatia; 8 St. Catherine Hospital, Zabok and Zagreb, Croatia; 9 Eberly College of Science, The Pennsylvania State University, University Park, PA, USA,; 10 University of Split, Medical School, Split, Croatia; 11 Children’s Hospital Srebrnjak, Zagreb, Croatia

## Abstract

**Aim:**

To present the results obtained in the identification of human remains from World War II found in two mass graves in Ljubuški, Bosnia and Herzegovina.

**Methods:**

Samples from 10 skeletal remains were collected. Teeth and femoral fragments were collected from 9 skeletons and only a femoral fragment from 1 skeleton. DNA was isolated from bone and teeth samples using an optimized phenol/chloroform DNA extraction procedure. All samples required a pre-extraction decalcification with EDTA and additional post-extraction DNA purification using filter columns. Additionally, DNA from 12 reference samples (buccal swabs from potential living relatives) was extracted using the Qiagen DNA extraction method. Quantifiler^TM^ Human DNA Quantification Kit was used for DNA quantification. PowerPlex ESI kit was used to simultaneously amplify 15 autosomal short tandem repeat (STR) loci, and PowerPlex Y23 was used to amplify 23 Y chromosomal STR loci. Matching probabilities were estimated using a standard statistical approach.

**Results:**

A total of 10 samples were processed, 9 teeth and 1 femoral fragment. Nine of 10 samples were profiled using autosomal STR loci, which resulted in useful DNA profiles for 9 skeletal remains. A comparison of established victims' profiles against a reference sample database yielded 6 positive identifications.

**Conclusion:**

DNA analysis may efficiently contribute to the identification of remains even seven decades after the end of the World War II. The significant percentage of positively identified remains (60%), even when the number of the examined possible living relatives was relatively small (only 12), proved the importance of cooperation with the members of the local community, who helped to identify the closest missing persons’ relatives and collect referent samples from them.

DNA analysis plays a key role in the identification of missing persons and victims of mass fatality incidents, and DNA profiling could be regarded as a core method for such identification (1). The primary value of this procedure significantly increased over the last twenty years due to the introduction of short tandem repeat (STR) loci in routine forensic testing, especially their optimized versions, miniSTRs or STR markers located at the Y and X chromosomes. Data obtained by DNA typing are highly reliable and can be used as a powerful tool that produces reliable results (2).

Various procedures could be used to identify human remains (3) and the choice of an appropriate procedure and its usefulness primarily depend on the condition of the remains. When it comes to skeletal remains from World War II (WWII), DNA analysis has already proved to be efficient, and in most cases, the only applicable approach to human identification (4,5). Furthermore, a seven-decade long soil deposition is a significant adverse factor, which is why most of the laboratories use optimized procedures in the analysis of these samples. Over the last 20 years, DNA identification of victims of the wars in Bosnia and Herzegovina (B&H) (6) and Croatia in the 1990s (3), the analysis of WWII skeletal remains in Slovenia (4,5), and the analysis of several centuries old archeological samples (7) successfully identified a number of human skeletal remains and overcame a number of challenges, such as optimization of DNA extraction protocols, specific laboratory procedures, communication with the relatives, etc. Here we once again utilized the experience gained from these projects to identify skeletal remains from the WWII mass graves in B&H.

There are no precise official data about the number of killed and missing persons from the WWII period in B&H, although this number is roughly estimated to about 180 000 (8). This estimate is even higher if we include the victims from immediate post-war incidents and mass executions by communist authorities. Most of those crimes were hidden for almost 70 years. For example, more than 2300 persons from the Ljubuški municipality alone were killed during WWII, and immediately after it, that is, in 1945. Almost 50% of the victims are still considered missing and their burial sites are not known (9).

As a consequence of continuous pressure from living relatives, the regional authorities and local communities recently put in significant efforts to identify individuals discovered in several mass graves scattered throughout the region. They commissioned a DNA analysis of skeletal remains and reference samples. Here, we report the results of the identification of the WWII victims from the city of Ljubuški, south Herzegovina.

## Materials and methods

### Handling of skeletal remains

**Figure 1:**
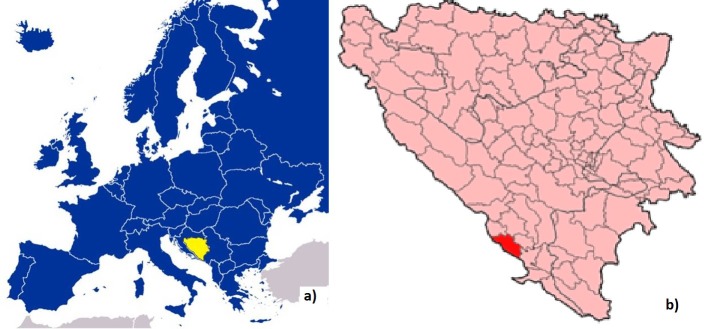
Location of (**A**) Bosnia and Herzegovina and (**B**) Ljubuški area.

More than 60 skeletal remains from different locations in and around Ljubuški city (south Bosnia and Herzegovina) were exhumed during 2010 and 2011 (Figure 1). Exhumations were initiated by family members as well as the local Commission for Marking and Maintenance of Graves from World War II and the Post-War Period in the municipality of Ljubuški. The work was performed by forensic experts from the University Clinical Center Split, Croatia. Three years later mortal remains of 10 persons, which were exhumed from the uncovered graves closest to the center of the city Ljubuški, were selected for DNA analysis. Testimonies from living relatives indicated that these remains belonged to the inhabitants of Ljubuški that were shot by communist forces in the spring of 1945. According to these testimonies, local people knew about this burial site, but it could not be marked until the end of the communist rule in B&H.

The remains were exhumed and samples for DNA analysis (femoral fragments and teeth) were collected and labeled. The samples (teeth and bone fragments from nine bodies and bone fragment from one body) were subsequently transported to Genos DNA laboratory in Zagreb and the Laboratory of Forensic Genetics at the Institute for Genetic Engineering and Biotechnology (INGEB) University of Sarajevo, where they were stored at -80°C until further processing.

### Sample preparation

For the bone sample, the entire exterior was sanded clean to remove potential contaminants. Bone surface was cleaned from remnant soft tissue and soil traces using a grinding stone attached to a Dremel^®^ rotary tool. The sample was then successively washed in mild detergent, 5% bleach, sterile distilled water, and 100% ethanol and subsequently air-dried. The thoroughly dried sample was pulverized using a sterilized Waring^®^ blender and powder and then transferred to sterile 15-mL conical polypropylene tubes. With the exception of the sanding phase, the same procedure was applied to teeth. An EDTA decalcification step was used for each of the samples. Also, the same extractions were performed for each sample following a previously described phenol-chloroform DNA extraction protocol (10). DNA analysis included teeth from 9 skeletons (laboratory code S1 to S9/03-14) and a femoral fragment from one skeleton (laboratory code S10/03-14) because of absence of teeth. Filter units were used for DNA purification and concentration. The concentrates were subsequently transferred to 1.5-mL microcentrifuge tubes and diluted with DNA-free ddH_2_O to a final volume of maximum 100 μL.

### DNA analysis

The DNA concentration was determined using Quantifiler Human DNA Quantification Kit (Applied Biosystems Foster City, CA, USA) (11). The reaction was carried out in the AB 7300 Real-Time PCR System (Applied Biosystems) according to manufacturer’s recommendations.

The PowerPlex^®^ESI kit (Promega Corp., Madison, WI, USA) was used to simultaneously amplify 15 STR loci: D3S1358, D8S1179, D18S51, D21S11, FGA, TH01, vWA, D2S441, D10S1248, D22S1045, D1S1656, D12S391, D2S1338, D16S539, D19S433 as well as the gender determination locus, Amelogenin. Amplification was carried out as described previously (12). The total volume of each reaction was 25 μL. Additionally, the PowerPlex^®^Y23 kit (Promega Corp.) was used to simultaneously amplify 23 Y-STR loci according to manufacturer’s recommendations (13). PCR amplification was carried out using the Applied Biosystems® GeneAmp® PCR System 9700 (Life Technologies) according to manufacturer’s recommendations. Electrophoresis of the amplification products was performed on an ABI PRISM 310 Genetic Analyzer (Applied Biosystems). The raw data were compiled and analyzed using 310 Data Collection Software and GeneMapper^TM^ 3.2.

### Handling of referent samples

Referent samples (12 buccal swabs) from potential living relatives were collected, recorded, and preliminarily labeled by local DNA experts. Living relatives were directly contacted by a local person (Mr Jure Lauc), who was present during the collection of each sample. Collection was performed with an Internal GENOS collection kit, and dried and labeled samples were transported to the laboratory. Upon arrival, the samples were relabeled and the relevant information entered into the Chain of Custody forms. The samples were stored at -80°C until DNA extraction was performed using the QIAmp DNA Mini Kit (14).

PowerPlex^®^ ESI kit (Promega Corp.) was used for further analysis. Similar amounts of DNA were used in all PCR reactions. Amplification was carried out as described previously (12). The total reaction volume was 10 μL. Additionally, PowerPlex^®^Y23 kit (Promega Corp.) was used to simultaneously amplify 23 Y-STR loci according to manufacturer’s recommendations (13). PCR amplification was carried out by the Applied Biosystems® GeneAmp® PCR System 9700 (Life Technologies) according to manufacturer’s recommendations. Electrophoresis of the amplification products was performed on an ABI PRISM 310 Genetic Analyzer (Applied Biosystems). The raw data were compiled and analyzed using 310 Data Collection Software and GeneMapper^TM^ 3.2.

### Statistical analysis

Comparative analysis of DNA profiles obtained from skeletal remains and referent samples, estimation of potential familiar relationships, calculation of paternity and sibling indexes, as well as calculation of matching probability were performed according to the previously used statistical approach (15).

## Results

Ten skeletal remains samples (9 teeth and 1 bone) were processed. The quantification results illustrated that the mean concentration of isolated DNA varied between 4.00 × 10^-3^ ng/μL and 7.00 × 10^−1^ ng/μL, for samples from which DNA profiles were obtained. Based on the quantification results, an extended PCR procedure (up to 32 cycles with an extended elongation time) was recommended for 3 samples.

A total of 9 DNA profiles were obtained, which means that profiling success rate was 90% (9/l0 remains; Table 1). The number of successfully analyzed loci per established STR profile varied from 12 to 16. Also, 12 referent buccal swabs collected from the missing persons’ relatives were 100% successfully profiled.

**Table 1:** Summary of DNA analysis of skeletal remains

Body code	Sample code	Collected samples	EDTA decalc. step	Polymerase chain reaction protocol	Successfully DNA profiled	Number of loci with detected alleles	Type of analysis	Positive identification	Identified by DNA profile from
*G2K1*	*S1/03-14*	Tooth/bone	+	Standard	Yes	14/16	Autosomal	No	-
*G1K2*	*S2/03- 14*	Tooth/bone	+	Extended	Yes	15/16	Autosomal	Yes	Daughter
*G1K6*	*S3/03- 14*	Tooth/bone	+	Standard	Yes	14/16	Autosomal/Y chromosomal	Yes	Living nephew/deceased brother
*G1K4*	*S4/03- 14*	Tooth/bone	+	Standard	Yes	15/16	Autosomal	Yes	Son
*G1K5*	*S5/03- 14*	Tooth/bone	+	Standard	Yes	14/16	Autosomal	Yes	Son
*G2K3*	*S6/03- 14*	Tooth/bone	+	Standard	Yes	12/16	Autosomal	No	-
*G1K1*	*S7/03- 14*	Tooth/bone	+	Standard	Yes	16/16	Autosomal	Yes	Daughter
*G1K3*	*S8/03- 14*	Tooth/bone	+	Standard	Yes	15/16	Autosomal	Yes	Son
*G2K6*	*S9/03- 14*	Tooth/bone	+	Extended	Yes	13/16	Autosomal	No	-
*G2K2*	*S10/03- 14*	Bone	+	Extended	No	3/16	Autosomal	No	-

Comparison of victims’ profiles against the referent database resulted in 6 positive identifications. All identified persons were men and first 5 were identified by autosomal DNA analysis, while 1 was identified by a combination of autosomal and Y-STR analysis. Five of the 6 identified persons were identified using the living children’s DNA profiles and 1 was identified using the living nephew’s DNA profile in combination with the established DNA profile of his deceased brother who was buried in the same grave and was one of the 5 previously mentioned identified persons (Table 1).

## Discussion

This study demonstrated that the experience gathered over the last two decades through identification of missing persons in this region could be almost routinely applied without significant modifications in the analysis of WWII skeletal remains. DNA analysis successfully identified remains even seven decades after the end of the World War II and proved to be the essential approach for the identification of remains from that period.

Genetic typing by STR loci analysis has over the last two decades (3-5) become a primary method of choice for the identification of human remains. The latest studies conducted on the WWII samples (4,5,16-19) were done using STR techniques instead of mtDNA, which used to be the mostly used identification method for the samples from that period (20).

Nowadays, application of improved old or completely new procedures increases the possibility for nuclear DNA profiling of degraded skeletal remains and gives us an opportunity to increase success of the skeletal remains DNA profiling routine procedures. Additionally, these results emphasize the importance of targeted, prompt, and efficient sample collection from living relatives. This approach yielded a 60% success identification rate, which is considerably higher than rate from our previous studies (4,5). The crucial reason for this success was the involvement of individuals from the local community who searched for victims’ close relatives. This is why we had so many samples from the direct offspring (daughters and sons) of the victims, which led to statistically satisfying and powerful results of the identification although the number of processed referent samples was small. This was something that our previous studies (4,5) lacked and that had a significant influence on the final success rate of identification.

The biggest limitation of this type of studies is the lack of clear strategy for identification of WWII victims. Therefore, we suggest that our strategy should be used as a model for further activities in this field, as well for additional DNA identification of human remains exhumed from the WWII mass graves in B&H and other neighboring countries. Once again it was proven that forensic science might bring closure to families who had been searching for their loved ones for decades. It is important to highlight that many of the closest relatives of the missing are aged and it is urgent to collect samples from them. It is safe to assume that without their DNA profile the entire identification process would be much more complicated if not impossible.
